# CD8^+^ T Cells Directed Against a Peptide Epitope Derived From Peptidoglycan-Associated Lipoprotein of *Legionella pneumophila* Confer Disease Protection

**DOI:** 10.3389/fimmu.2020.604413

**Published:** 2020-12-08

**Authors:** Sun Jin Kim, Jeong-Im Sin, Min Ja Kim

**Affiliations:** ^1^ Department of Medicine, College of Medicine, Korea University, Seoul, South Korea; ^2^ Department of Microbiology, School of Medicine, Kangwon National University, Chuncheon, Gangwon-do, South Korea; ^3^ Division of Infectious Diseases, Department of Internal Medicine, Korea University Anam Hospital, Korea University College of Medicine, Seoul, South Korea; ^4^ Institute of Emerging Infectious Diseases, Korea University College of Medicine, Seoul, South Korea

**Keywords:** *Legionella pneumophila*, peptidoglycan-associated lipoprotein, peptide epitope, cytotoxic T-lymphocyte, adaptive immunity

## Abstract

*Legionella pneumophila*, an intracellular bacterium, may cause life-threatening pneumonia in immunocompromised individuals. Mononuclear cells and antibodies have been reported to be associated with the host defense response against *L. pneumophila.* This study is to determine whether *Legionella* peptidoglycan-associated lipoprotein (PAL)-specific CD8^+^ T cells are directly associated with protection against *L. pneumophila*, with a focus on potential epitopes. Synthetic peptides derived from PAL of *L. pneumophila* were obtained and tested through in *vitro* and *in vivo* cytotoxic T lymphocyte (CTL) assays for immunogenicity. PAL DNA vaccines or a peptide epitope with or without CpG-oligodeoxynucleotides (ODN) was evaluated for protection against *L. pneumophila* infection in animal models. When mice were immunized with DNA vaccines expressing the PAL of *L. pneumophila*, they were significantly protected against a lethal challenge with *L. pneumophila* through induction of antigen-specific CD8^+^ CTLs. Of the 13 PAL peptides tested, PAL_92-100_ (EYLKTHPGA) was the most immunogenic and induced the strongest CTL responses. When mice were immunized with the PAL_92-100_ peptide plus CpG-ODN, they were protected against the lethal challenge, while control mice died within 3–6 days after the challenge. Consistent with lung tissue histological data, bacterial counts in the lungs of immunized mice were significantly lower than those in control mice. Also, the amino acid sequence of PAL_92-100_ peptides is conserved among various *Legionella* species. To our knowledge, this study is the first to demonstrate that PAL_92-100_-specific CD8^+^ T cells play a central role in the host defense response against *L. pneumophila*.

## Introduction

*Legionella pneumophila* is the causative pathogen of a severe form of pneumonia, Legionnaires' disease, with high mortality and morbidity. The *L. pneumophila* bacterium is a Gram-negative facultative intracellular pathogen, which is commonly found in the natural environment and in immunocompromised individuals (1–4). Whether sporadic, epidemic, nosocomial, or community-acquired, Legionnaires’ disease can be deadly, especially among patients with reduced immune competence. *L. pneumophila* enters the human respiratory tract as a result of inhalation of aerosols from a contaminated water source, and thereafter infects human alveolar macrophage and lung epithelial cells (5–8).

Cell-mediated immunity, but not humoral immunity, appears to play an important role in the host defense response against *L. pneumophila* (9–11). In human studies, activated mononuclear cells inhibited the intracellular multiplication of *L. pneumophila* (9, 11). Moreover, alveolar macrophages were suggested to be an effector cell acting to inhibit bacterial multiplication (11). In animal models, antibodies were also associated with protection during early stages of airway infection (12). Similarly, immunization with *L. pneumophila* membranes resulted in induction of strong cellular immune responses and protective immunity against a lethal challenge with *L. pneumophila* (13). In addition, the major secretory and outer membrane proteins of *L. pneumophila* were reported to be effective at inducing protective immunity against *L. pneumophila* (14, 15).

The 19-kDa peptidoglycan-associated lipoprotein (PAL) is an outer membrane lipoprotein that is conserved among various *Legionella* species; in 1991, PAL was sequenced and characterized as the most prominent *Legionella* surface antigen (16). As PAL has been found in the urine of infected patients, it has also been used as a diagnostic antigen for legionellosis (17, 18). PAL activates murine macrophages through Toll-like receptor (TLR) 2-mediated signaling, which stimulates the released of pro-inflammatory cytokines, such as IL-6 and TNF-α (19). Immunization with a full-length 528-bp *pal* gene vaccine induced IFN-γ and IL-2 production from spleen cells, as well as potent cytotoxic T lymphocyte (CTL) responses (20). Recombinant PAL (rPAL) also induced protective immunity against *L. pneumophila* infection (21). Together, the results of these studies suggest that PAL may be a potential vaccine target for prevention of *L. pneumophila* infection. In our animal study, PAL DNA and rPAL vaccines induced antigen-specific antibody and CTL responses (20). However, it is still unclear whether PAL-specific antibody or the CD8^+^ CTL response is mainly responsible for protecting animals from *Legionella* infection.

In this study, we demonstrated that PAL-specific CD8^+^ CTLs were responsible for protection from infection with *L. pneumophila*. Among 13 peptide candidates derived from the *L. pneumophila* PAL, one peptide (PAL_92-100_) was recognized by PAL-specific CD8^+^ T cells. Immunization with the PAL_92-100_ peptide resulted in the induction of antigen-specific CD8^+^ CTL responses, improved survival, and reduced lung bacterial burden after *L. pneumophila* infection. Thus, this study clearly demonstrates that PAL_92-100_-specific CD8^+^ CTLs mediate anti-*Legionella* protective immunity, and that peptides containing a well-conserved PAL epitope may be effective vaccines against various *Legionella* species.

## Materials and Methods

### Prediction of Class I MHC Binding Epitopes 

Peptides derived from the PAL of *L. pneumophila* serogroup 1 were designed using three Class I MHC binding molecule prediction programs, RANKPEP (http://bio.dfci.harvard.edu/RANKPEP), BIMAS (http://bimas.cit.nih.gov), and SYFPEITHI (http://syfpeithi.de). The programs were used to predict the binding activity of each peptide to Class I MHC haplotypes from BALB/c mice. The following selection criteria were used. First, 9-mer sequences with a high Class I MHC binding score were pre-selected from the full-length *Legionella* PAL sequence. Next, the peptides with the best Class I MHC binding scores were selected from within the entire sequence and were ranked according to the Class I MHC binding score for each online algorithm. Finally, the results from all algorithms were combined (consensus prediction).

### Synthetic Peptides 

The PAL peptides were synthesized by Sigma-Aldrich (St. Louis, MO, USA) and PEPTRON (Daejeon, Korea). The purity of peptide was synthesized to over 90%. The synthetic peptide amino acid sequences were as follows: PAL_1-9_ (MKAGSFYKL: P1), PAL_4-24_ (GSFYKLGLLVASAVLVAACS: P2), PAL_37-47_ (DGDATAQGL: P3), PAL_55-63_ (EPGESYTTQ: P4), PAL_65-73_ (PHNQLYLFA: P5), P_76-84_ (DSTLASKYL: P6), PAL_86-94_ (SVNAQAEYL: P7), PAL_92-100_ (EYLKTHPGA: P8), PAL_97-105_ (HPGARVMIA: P9), PAL_112-119_ (GSREYNVA: P10), PAL_124-132_ (RADTVAEIL: P11), PAL_135-147_ (AGVSRQQIRVVSY: P12), PAL_163-171_ (AQNRRVEFI: P13), as shown in **Table 1**.

**Table 1:** Predicted MHC class I-restricted peptides derived from peptidoglycan-associated lipoprotein of *Legionella pneumophila*.

Peptide No.	Position	Peptide Sequence	Haplotype
P1	PAL_1-9_	MKAGSFYKL	H-2L^d^
P2	PAL_4-24_	GSFYKLGLLVASAVLVAACSK	H-2L^d^
P3	PAL_37-45_	DGDATAQGL	H-2D^d^
P4	PAL_55-63_	EPGESYTTQ	H-2D^d^
P5	PAL_65-73_	PHNQLYLFA	H-2L^d^
P6	PAL_76-84_	DSTLASKYL	H-2L^d^
P7	PAL_86-94_	SVNAQAEYL	H-2L^d^
P8	PAL_92-100_	EYLKTHPGA	H-2K^d^
P9	PAL_97-105_	HPGARVMIA	H-2L^d^
P10	PAL_112-119_	GSREYNVA	H-2L^d^
P11	PAL_124-132_	RADTVAEIL	H-2K^d^
P12	PAL_135-147_	AGVSRQQIRVVSY	H-2K^d^
P13	PAL_163-171_	AQNRRVEFI	H-2D^d^

### Bacteria

*L. pneumophila* strain Philadelphia-1 (ATCC 33152), an isolate from the lung tissue of a Legionnaires’ disease patient from Philadelphia, Pennsylvania (32), was tested in this study. Bacteria were cultured from frozen stock on buffered charcoal yeast extract (BCYE-α) agar plates supplemented with L-cysteine, ferric pyrophosphate, and α-ketoglutaric acid, incubated at 37°C with 5% CO_2_ for 72 h. The bacteria were maintained at –80°C before use in infection.

### Experimental Animals

Female BALB/c (H-2^d^) mice, 6 to 8 weeks of age, were purchased from Oriental Bio Inc. (Chungbuk, Korea).

### Immunization of Mice

Mice were immunized with PAL plasmid DNAs (pcDNA3-PAL) (20) or synthetic PAL peptides. For DNA immunization, 100 µg of pcDNA3-PAL was injected into the tibialis anterior muscle of both legs and the mice received booster injections at the same dose at 1-week intervals. For synthetic peptide immunization, mice were immunized subcutaneously (s.c.) with 20 µg of PAL peptides plus 20 µg of CpG-oligodeoxynucleotide (ODN) in 100 µl of phosphate-buffered saline (PBS). They mice received booster injections at the same dose at 1-week intervals. The CpG-ODN (5’-TCCATGACGTTCCTGACGTT-3’) containing a phosphorothioate backbone was purchased from GenoTech, Daejeon, Korea.

### *In Vivo* Depletion of CD8^+^ T Cells 

Anti-CD8 IgGs (100 µg) were injected intraperitoneally (i.p.) into mice on the indicated days. A hybridoma cell line (clone 2.43) was purchased from the American Type Culture Collection (Manassas, VA, USA), and anti-CD8 IgGs were purified as previously described (22). Control IgGs were purchased from Sigma-Aldrich (St. Louis, MO, USA). Anti-CD8 IgG administration resulted in more than 98% depletion of CD8^+^ T cell at 3–5 days following antibody treatment.

### Measurement of Cytokine Production 

Cytokine (IFN-γ and TNF-α) concentrations were measured by ELISA. The splenocytes were incubated at 37^o^C with/without antigens. Cytokine concentrations in the cell culture supernatants were measured using IFN-γ (BD Biosciences, San Jose, CA, USA) and TNF-α (BioLegend, San Diego, CA, USA) ELISA kits according to the manufacturer’s instructions. The analyses were completed in triplicate, and cytokine concentrations were calculated by regression analyses of a standard curve.

### *In Vitro* CTL Assay 

Splenocytes were collected 1 week after the final immunization and mixed with 2 × 10^6^ naive splenocytes that had been previously treated with mitomycin C and cultured in the presence of P8 peptides (5 μg/ml) in a 24-well plate for 5 days at 37°C. The cells were washed twice with complete RPMI 1640 and then used as effector cells. Syngeneic naive splenocytes were prepared by adsorption of P8 peptides (5 μg/ml) and rPAL (5 µg/ml) for 3 days at 37°C, washed three times with complete RPMI 1640, and resuspended at a concentration of 5 × 10^6^ cells per ml for use as target cells. The pulsed target (T) cells (1 × 10^4^ cells/well) were added to a 96-well plate, and effector cells (E) were then added a E:T ratios of 50:1, 30:1, or 10:1. After incubation for 4 h, antigen-specific lysis was measured using the CytoTox 96^®^ Non-Radioactive Cytotoxic Assay (Promega, Madison, WI, USA) in accordance with the manufacturer’s instructions. The percent specific lysis was calculated as follows: % specific lysis = 100 × (experimental − spontaneous) / (maximal − spontaneous).

### *In Vivo* CTL Assay 

Splenocytes from naïve mice were incubated with 5 µg/ml of P8 peptides at 37°C for 90 min. They were prepared by being divided into two tubes containing 2 × 10^7^ cells/ml in RPMI-1640 with 2.5% FBS, and the fluorescent carboxylfluorescein diacetate succinimidyl ester (CFSE) dye (BD Bioscience) added at 2.5 µM (CFSE^low^) or 20 µM (CFSE^high^), then the cells were resuspended and incubated at 37°C for 40 min. The stained cells were washed two times with PBS. Each mouse received an intravenous injection of a mixture of 1 × 10^7^ CFSE^low^ and 1 × 10^7^ CFSE^high^ cells in a total volume of 200 µl of RPMI 1640 without serum. After 18 h, mice were sacrificed in a CO_2_ chamber, and the spleens were removed and processed for flow cytometry. The percent lysis was calculated as [100 × (1 − [γ_unprimed_/γ_primed_])]. The γ (ratio) was calculated as %CFSE^low^/%CFSE^high^.

### Intravenous and Intranasal Challenges With *L. pneumophila*

Mice were challenged intravenously (i.v.) with 100 μl of a bacterial suspension containing 2 × 10^7^ CFU of *L. pneumophila.* Mice were also challenged intranasally (i.n.) with 40 μl of bacterial suspension containing 1 × 10^9^ CFU of *L. pneumophila.* In this case, mice were administered cyclophosphamide (75 mg/kg or 150 mg/kg) every day for 3 days prior to intranasal challenge. The approximate number of bacteria was estimated by measuring the absorbance at 600 nm (1 OD value at 600 nm was assumed as 1 × 10^9^ CFU/ml). Survival of infected mice was assessed daily for 10–14 days following the bacterial challenge. Percent survival was calculated as [the number of dead mice/the number of all tested mice × 100].

### Bacterial Burden Assay

Mice were challenged i.n. with *L. pneumophila.* Forty-eight hours after the challenge, the mice were sacrificed and the lungs removed and homogenized in sterile PBS using a tissue homogenizer (Pyrex Corning, Greencastle, PA, USA). Ten-fold serial dilutions of the lung homogenates were plated on BCYE-α agar containing cefamandole, polymyxin B, and vancomycin. The bacteria were cultured for 72 to 96 h at 37°C in 5% CO_2_ for determination of the number of viable *L. pneumophila*.

### Histological Analyses 

After an intranasal challenge with *L. pneumophila*, mice were sacrificed and the lungs were harvested for histopathologic measurements. Mouse lungs were fixed using 4% paraformaldehyde for 48 h, dehydrated, and embedded in paraffin. The sections (3 μm) were stained with hematoxylin and eosin (H&E) to visualize inflammatory cells infiltrating the lungs.

### Statistical Analyses 

All statistical analyses were performed by one-way analysis of variance (ANOVA) with *post-hoc* Dunnett’s test and chi-square test (Fisher’s exact test) using the SPSS13.0 program. Unless noted, ANOVA was used. The values of the experimental groups were compared with the values of the control group. Any *p* values <0.05 were considered to be significant.

## Results

### Major Roles of CD8^+^ T Cells in Protection From *L. pneumophila* Infection and Identification of the Class I MHC Epitopes 

**Figure 1:**
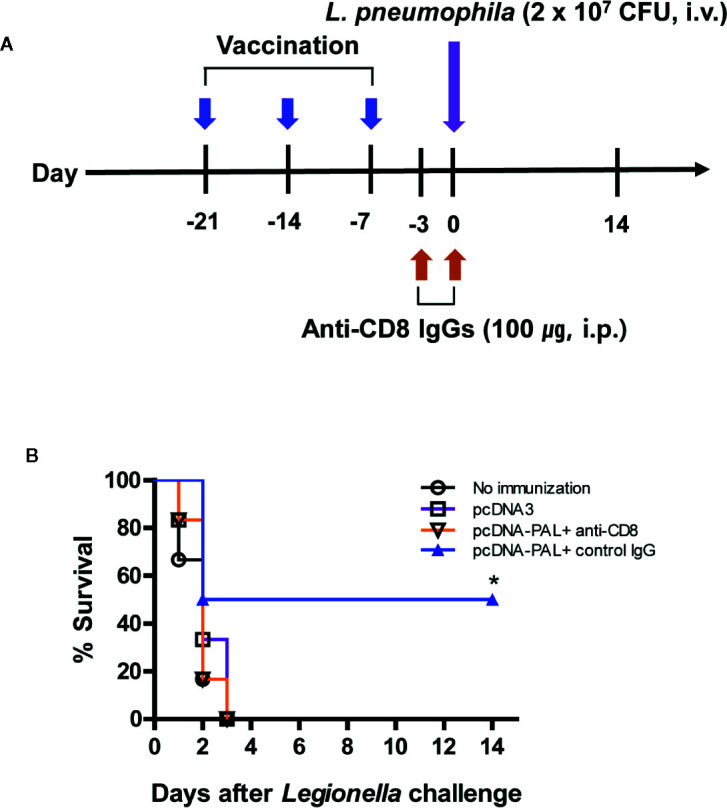
PAL DNA vaccination, CD8+ T cell depletion, and survival of mice after lethal challenge with *L. pneumophila*. **(A)** Schematic diagram showing PAL DNA vaccination, CD8+ T cell depletion, and bacterial challenge. Each group of mice (*n* = 6/group) was immunized intramuscularly with 100 µg of pcDNA3-PAL at 0, 1, and 2 weeks. At 3 weeks, the mice were challenged intravenously (i.v.) with *L. pneumophila* at 2 × 10^7^ CFU per mouse. For depletion of CD8+ T cell subsets, the animals were injected with 100 µg of anti-CD8 antibody and control IgGs on days −3 and 0 of bacterial challenge. **(B)** Percent survival. Surviving animals from bacterial challenge were counted at the indicated time points.

**Figure 2:**
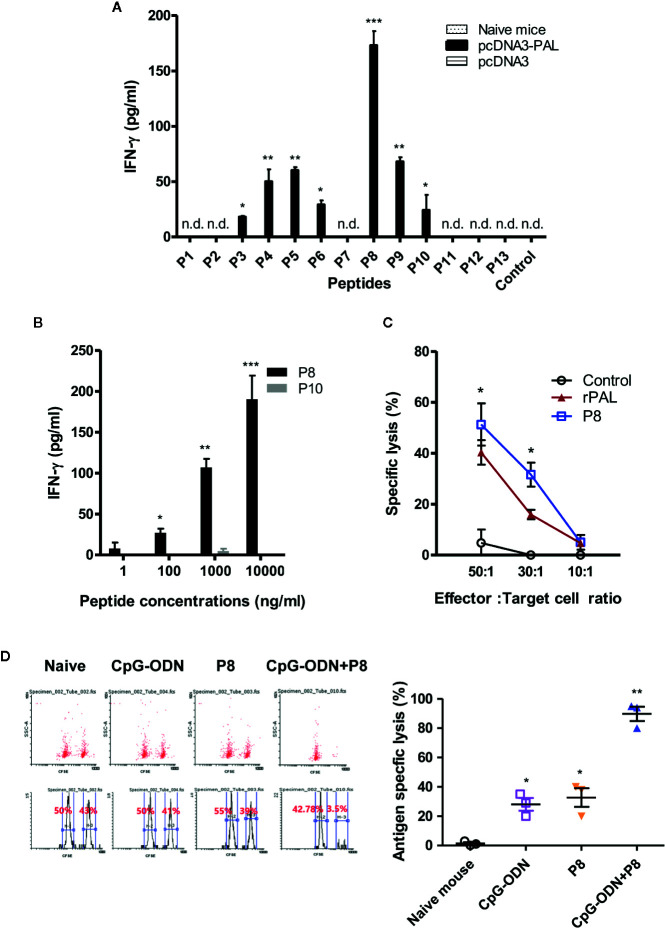
IFN-γ and CTL responses to PAL peptides. **(A)** To determine which peptides were able to induce IFN-γ production from PAL-specific spleen cells, mice were immunized with pcDNA3-PAL at 0, 1, and 2 weeks. At 3 weeks, the mice were sacrificed and the spleens were harvested. Six × 10^6^ splenocytes were stimulated for 2 days at 37^o^C with each of 13 peptides (P1–P13) at a final concentration of 5 µg/ml. The cell culture supernatants were collected and IFN-γ concentrations measured. n.d. (not detectable). *p < 0.05 compared to control, **p < 0.05 compared to P6, ***p < 0.05 compared to P9. **(B)** We repeated the above experiments, except that the splenocytes were stimulated with P8 or P10 (as a control) at final concentrations of 1, 200, 1,000, or 10,000 ng/ml. *p < 0.05 compared to 1 ng/ml, **p < 0.05 compared to 100 ng/ml, ***p < 0.05 compared to 1,000 ng/ml. **(C)** Mice were immunized and the spleens were obtained as above. *In vitro* lytic activity was measured using the splenocytes as effector cells and syngeneic targets (primed with P8 or rPAL) in the LDH release cytotoxicity assay, as described in the *Methods and Materials*. *p < 0.05 compared to control. **(D)** A CFSE-based cytotoxicity assay was performed to measure *in vivo* lytic activity, as described in the *Methods and Materials*. Cells with low and high density CFSE staining were gated and the CFSE intensity, as assessed by flow cytometry, was plotted. One representative result (% lysis) is shown. The values and bars represent mean IFN-γ concentrations and percent lysis and the SDs, respectively. *p < 0.05 compared to naïve mouse, **p < 0.05 compared to CpG-ODN or P8.

We previously reported that both PAL DNA and rPAL vaccines induce antigen-specific antibody and CTL responses (20). It was also reported that rPAL confers protective immunity against a lethal dose of *L. pneumophila* challenge (21). In this study, we used the PAL DNA vaccine model to determine whether CD8^+^ T cells were responsible for protection from a lethal challenge with *L. pneumophila*. For this test, animals were immunized with PAL DNA vaccines and challenged i.v. with *L. pneumophila*, in the presence of CD8^+^ T cell depletion (**Figure 1A**). After the lethal challenge, 50% of control mice immunized with PAL DNA vaccines survived, however, survival rates of mice depleted of CD8^+^ T cells were 0%, similar to naïve control mice (**Figure 1B**). This result suggests that CD8^+^ T cells are indeed responsible for protection against *L. pneumophila*. Next, we determined which peptides of PAL proteins might be recognized by PAL-specific CD8^+^ T cells. For these experiments, we predicted CD8^+^ T cell-specific epitopes from 176 amino acid residues of the full-length *pal* gene of *L. pneumophila* using three Class I MHC binding molecule prediction programs (RANKPEP, BIMAS, and SYFPEITHI software). The peptides were selected based upon their binding affinity for the Class I MHC haplotypes (H-2L^d,^ H-2D^d^, H-2K^d^) of BALB/c mice and their amino acid length (a 9-mer). To this end, we obtained 13 peptides with a high Class I MHC binding scores, among which 11 peptides were 9-mers while two others, PAL_4-24_ (P2) and PAL_135-147_ (P12), had more than nine amino acids (**Table 1**). To determine which peptides might be recognized by PAL-specific CD8^+^ T cells, we used each of the 13 peptides to stimulate spleen cells (containing PAL-specific CD8^+^ T cells) from mice immunized with PAL plasmid DNAs. The IFN-γ concentrations in the cell culture supernatants were assessed. As seen in **Figure 2A**, P8 peptides induced the greatest IFN-γ production among the peptides tested. The other 12 peptides showed some or little induction of IFN-γ production. To confirm this result, we again stimulated the immune cells with an increasing dose of P8 peptides in parallel with P10 peptides as a control. As shown in **Figure 2B**, P8 peptides increased IFN-γ production in a concentration-dependent fashion, as opposed to P10 peptides which induced little IFN-γ production. Therefore, these results reveal that PAL-specific CD8^+^ T cells can recognize the P8 (PAL_92-100_) peptide in conjunction with Class I MHC molecules expressed on the cells from BALB/c mice.

### *In Vitro* and *In Vivo* CTL Responses to P8 Peptides

To investigate whether P8 (PAL_92-100_) peptides might increase PAL-specific CTL populations, we immunized mice with PAL DNA vaccines and obtained the spleen cells, which were stimulated *in vitro* with P8 peptides. These cells were used as effector cells against target cells primed with either P8 peptides or rPAL in an *in vitro* CTL assay. As shown in **Figure 2C**, a significantly greater degree of CTL activity was directed toward target cells that had been primed with P8 and rPAL, as compared to unprimed control target cells. In particular, CTL activity toward target cells that had been primed with P8 was 11% greater than for target cells primed with rPAL at an effector to target cell ratio of 50:1 (51% for P8 *vs.* 40% for rPAL). This result suggests that as an antigen, P8 (PAL_92-100_) peptide can stimulate PAL-specific CD8^+^ CTL cell populations, thereby enhancing their target cell killing activity *in vitro*. Next, we evaluated whether P8 peptides could induce antigen-specific CTL responses *in vivo*. As seen in **Figure 2D**, the groups of mice immunized with P8 plus CpG-ODN had dramatically greater CTL lytic activity than those immunized with either the P8 peptide or CpG-ODN alone. For example, P8-plus-CpG-ODN-immunized animals displayed 98% lytic activity. However, control mice and the groups immunized with either P8 or CpG-ODN alone had similar lytic activity (**Figure 2D**). In this study, a TLR9 agonist CpG-ODN was used as a peptide vaccine adjuvant. It has been reported that CpG-ODN elicits antigen-specific CTL responses when co-injected with proteins or peptides (as an immunogen) (23, 24). Collectively, these data indicate that the P8 (PAL_92-100_) peptide can induce and stimulate antigen-specific CD8^+^ CTL responses *in vitro* and *in vivo*.

### Survival of Mice Immunized With P8 Plus CpG-ODN After Lethal Intravenous or Intranasal Challenge With *L. pneumophila*

**Figure 3:**
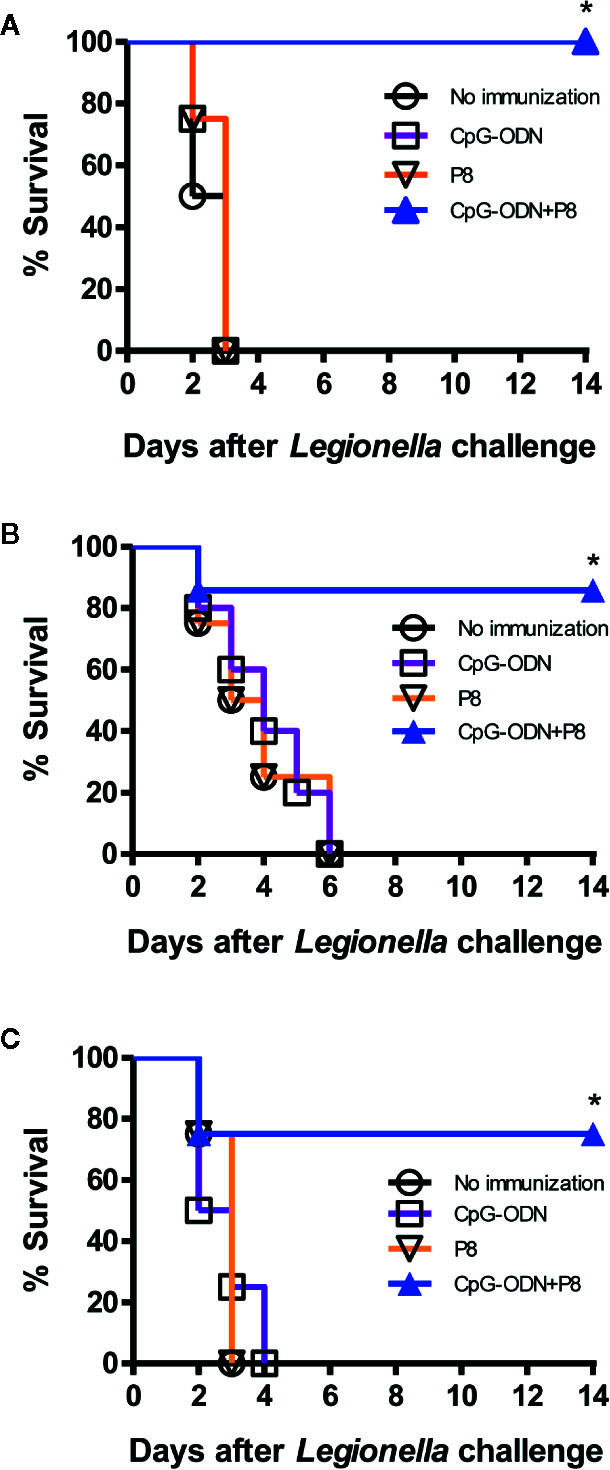
Survival of mice immunized with P8 plus CpG-ODN prior to lethal intravenous or intranasal challenge with *L. pneumophila*. **(A)** Each group of mice (*n* = 4/group) was immunized s.c. with P8 plus CpG-ODN at 0, 1, and 2 weeks. At 3 weeks, the mice were challenged i.v. with *L. pneumophila* at 2 × 10^7^ CFU per mouse. Surviving mice were counted at the indicated time points. **(B, C)** Each group of mice (*n* = 7 per group) was immunized as above. At 3 weeks, the mice were treated i.p. with 75 mg/kg **(B)** or 150 mg/kg **(C)** of cyclophosphamide every day for 3 days. The next day, the mice were challenged i.n. with *L. pneumophila* at 1 × 10^9^ CFU per mouse. Surviving mice were counted at the indicated time points. *p < 0.05 using Chi-square test compared to non-immunization.

To investigate whether P8 (PAL_92-100_) peptides improve survival after *Legionella* infection, we immunized mice with P8 plus CpG-ODN, followed by a lethal intravenous challenge with 2 × 10^7^ CFU of *L. pneumophila*. As seen in **Figure 3A**, the mouse groups immunized with P8 plus CpG-ODN had 100% survival after the lethal challenge, while the mouse groups immunized with either P8 or CpG-ODN alone, as well as naïve control groups, died within 3 days after the challenge. *L. pneumophila* infects humans through the respiratory tract and most frequently causes disease in immunosuppressed patients (5, 6). Therefore, we evaluated the protective efficacy of P8 peptides against bacterial infection when immunosuppressed animals were challenged i.n. with *L. pneumophila*. Cyclophosphamide has been used previously to render animals immunosuppressed and more susceptible to challenge with *L. pneumophila* (25). Cyclophosphamide is an alkylating chemotherapeutic agent, and as a cytotoxic drug has immunosuppressive effects (26, 27). In addition, lymphocyte counts were reported to reach a nadir four days after treatment with 150 mg/kg of cyclophosphamide (28). In consideration of these findings, immunized mice were administered a low (75 mg/kg of body weight) or high (150 mg/kg of body weight) dose of cyclophosphamide prior to intranasal challenge with *L. pneumophila*. When the groups immunized with P8 plus CpG-ODN were administered 75 mg/kg of cyclophosphamide, 85.7% of mice were alive 14 days after the intranasal challenge (1 × 10^9^ CFU per mouse; **Figure 3B**). However, the mouse groups immunized with either P8 or CpG-ODN alone, as well as the naïve control group died within 6 days after the challenge. When the groups immunized with P8 plus CpG-ODN were treated with 150 mg/kg of cyclophosphamide, 75% of mice were alive 14 days after the intranasal challenge (**Figure 3C**). However, the mouse groups immunized with either P8 or CpG-ODN alone, as well as naïve control groups died within 4 days after the challenge. Taken together, these data suggest that P8 (PAL_92-100_) peptides can induce resistance to *Legionella* infection in mice, with 75–100% survival, even in immunosuppressed animals after otherwise lethal infection with *L. pneumophila*.

### Bacterial Burdens in the Lungs of Mice Immunized With P8 Plus CpG-ODN and Cytokine Production by Spleen Cells After a Lethal Challenge With *L. pneumophila*

**Figure 4:**
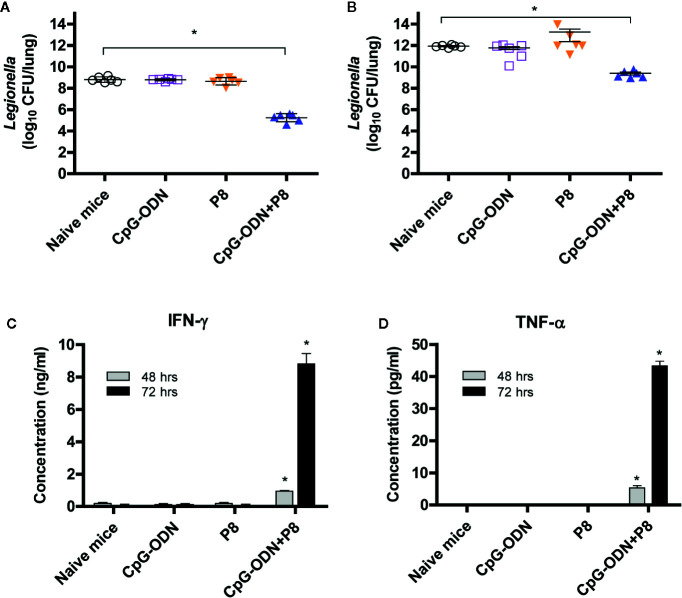
Both bacterial burden and cytokine induction in spleen cells of mice after lethal intravenous or intranasal challenge with *L. pneumophila*. **(A)** Each group of mice (*n* = 6/group) was immunized s.c. with P8 plus CpG-ODN at 0, 1, and 2 weeks. At 3 weeks, the mice were challenged i.v. with *L. pneumophila* at 2 × 10^7^ CFU per mouse. The mice were sacrificed 48 h post-challenge, the lungs were harvested, and the number of viable bacteria in the lung tissues was determined. **(B)** Each group of mice (*n* = 6/group) was immunized as above. At 3 weeks, the mice were administered 150 mg/kg of cyclophosphamide i.p. every day for 3 days. The next day, the mice were challenged i.n. with 1 × 10^9^ CFU of *L. pneumophila*. The mice were sacrificed 48 h post-challenge, the lungs were removed, and viable bacteria from the lung tissue were counted. **(C, D)** Each group of mice (*n* = 3/group) was immunized and administered cyclophosphamide, as described in panel B. The mice were sacrificed 16 h following the intranasal challenge and the spleens removed. The splenocytes were stimulated *in vitro* for 48–72 h at 37°C and the cell supernatants were collected for measurement of IFN-γ **(C)** and TNF-α **(D)**. *p < 0.05 compared to naïve mice.

We next tested whether P8 (PAL_92-100_) peptides might be able to reduce the bacterial burden in the lungs after *Legionella* infection. For this test, animals were immunized with P8 plus CpG-ODN, and challenged i.v. with *L. pneumophila*. As seen in **Figure 4A**, bacterial counts in the lung tissues of P8+CpG-ODN-immunized mice were reduced approximately 200-fold compared to control groups (naïve control and mice immunized with either P8 or CpG-ODN). A similar result was obtained when mice were challenged i.n. with *L. pneumophila* in the presence of immune suppression due to cyclophosphamide administration (150 mg/kg; **Figure 4B**). These findings are consistent with survival rates we observed previously. It is likely that the reduction in bacteria counts is mediated by antigen-specific CD8^+^ CTLs that are elicited by immunization with P8 plus CpG-ODN. We also measured IFN-γ and TNF-α production of the spleen cells from immunized mice administered cyclophosphamide (150 mg/kg). As seen in **Figures 4C, D**, spleen cells from the mice immunized with P8 plus CpG-ODN produced significantly more IFN-γ (C) and TNF-α (D) than cells from mice immunized with either P8 or CpG-ODN, as well as negative control mice producing a basal level of cytokines. It is notable that this cytokine production was measured in the absence of any antigen stimulation *in vitro*, suggesting that the cytokines were likely released from PAL_92-100_-specific spleen cells under stimulation with prior intranasal exposure to *L. pneumophila*. Therefore, these results demonstrate that P8 (PAL_92-100_) peptides can induce cytokine responses even in immunosuppressed animals.

### Histological Analyses of Mouse Lung Tissues After *L. pneumophila* Infection

**Figure 5:**
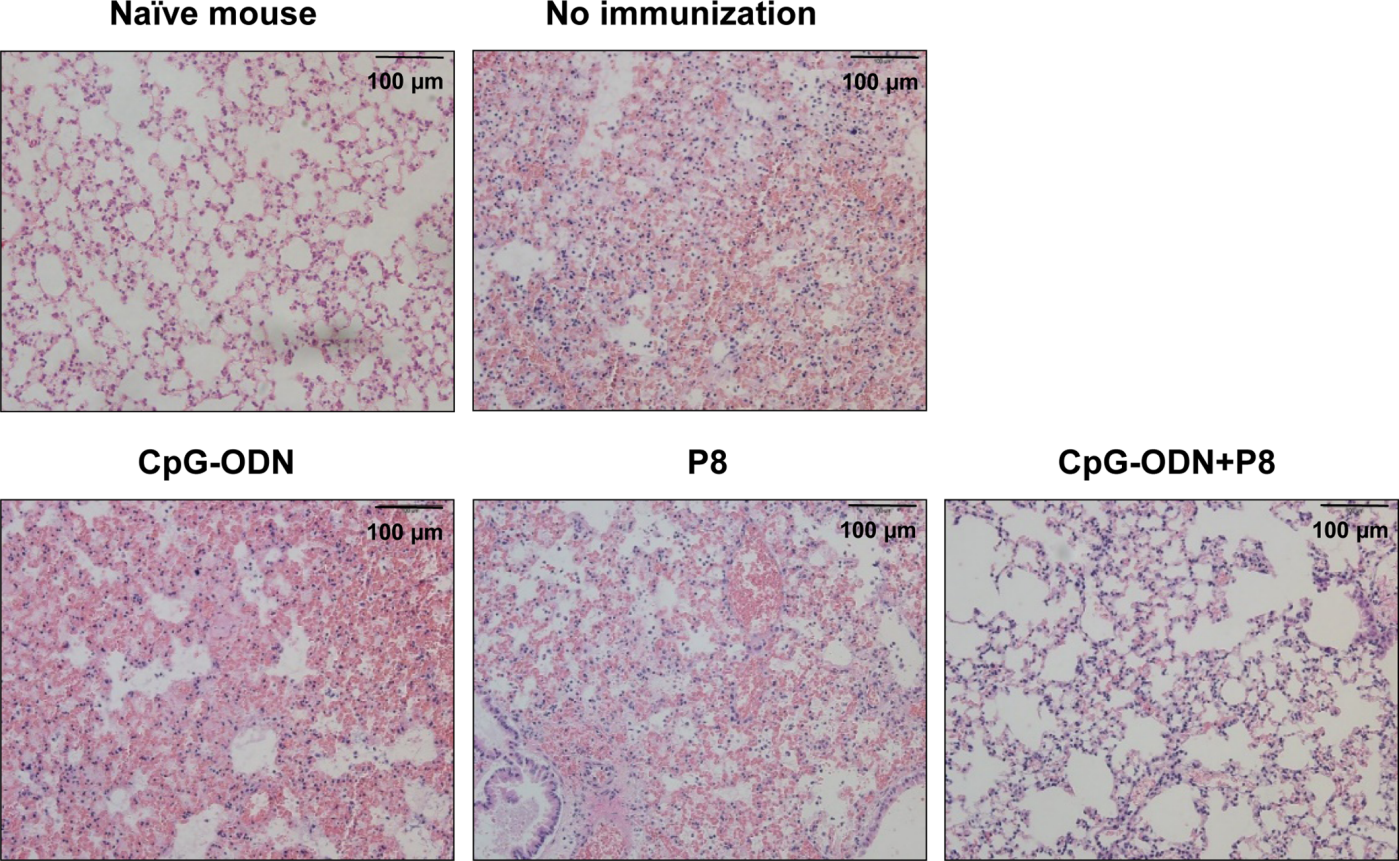
Histopathology of the lungs of mice challenged with a lethal dose of *L. pneumophila*. Each group of mice (*n* = 3/group) was immunized s.c. with P8 plus CpG-ODN at 0, 1, and 2 weeks. At 3 weeks, the mice were treated i.p. with 150 mg/kg of cyclophosphamide every day for 3 days. Next day, the mice were challenged i.n. with 1 × 10^9^ CFU of *L. pneumophila*. Upon animal death after the challenge, the lungs were removed from the mice and the lung tissues were sectioned, followed by staining with hematoxylin-eosin. Cell nuclei were stained dark blue, and cytoplasm were pink. Representative images of inflammatory lesions are shown (magnification, × 200).

To compare histological changes in the lungs of mice immunized with P8 (PAL_92-100_) plus CpG-ODN following infection with *L. pneumophila*, the lungs were harvested and stained with H&E. Hemorrhage, destruction of alveolar tissue, hyperplasia of alveolar walls, interstitial edema, and infiltration of numerous inflammatory cells were evident in lung tissues from the control group and groups immunized with either P8 or CpG-ODN alone (**Figure 5**). However, significant reductions in inflammatory infiltration in alveolar and interstitial space were noted in the groups immunized with P8 plus CpG-ODN. These data suggest that animals immunized with P8 plus CpG-ODN alone can protect against lung tissue damage resulting from infection with *L. pneumophila*.

### Presence of Conserved P8 Peptide Region in the PAL of *Legionella* Species 

**Figure 6:**
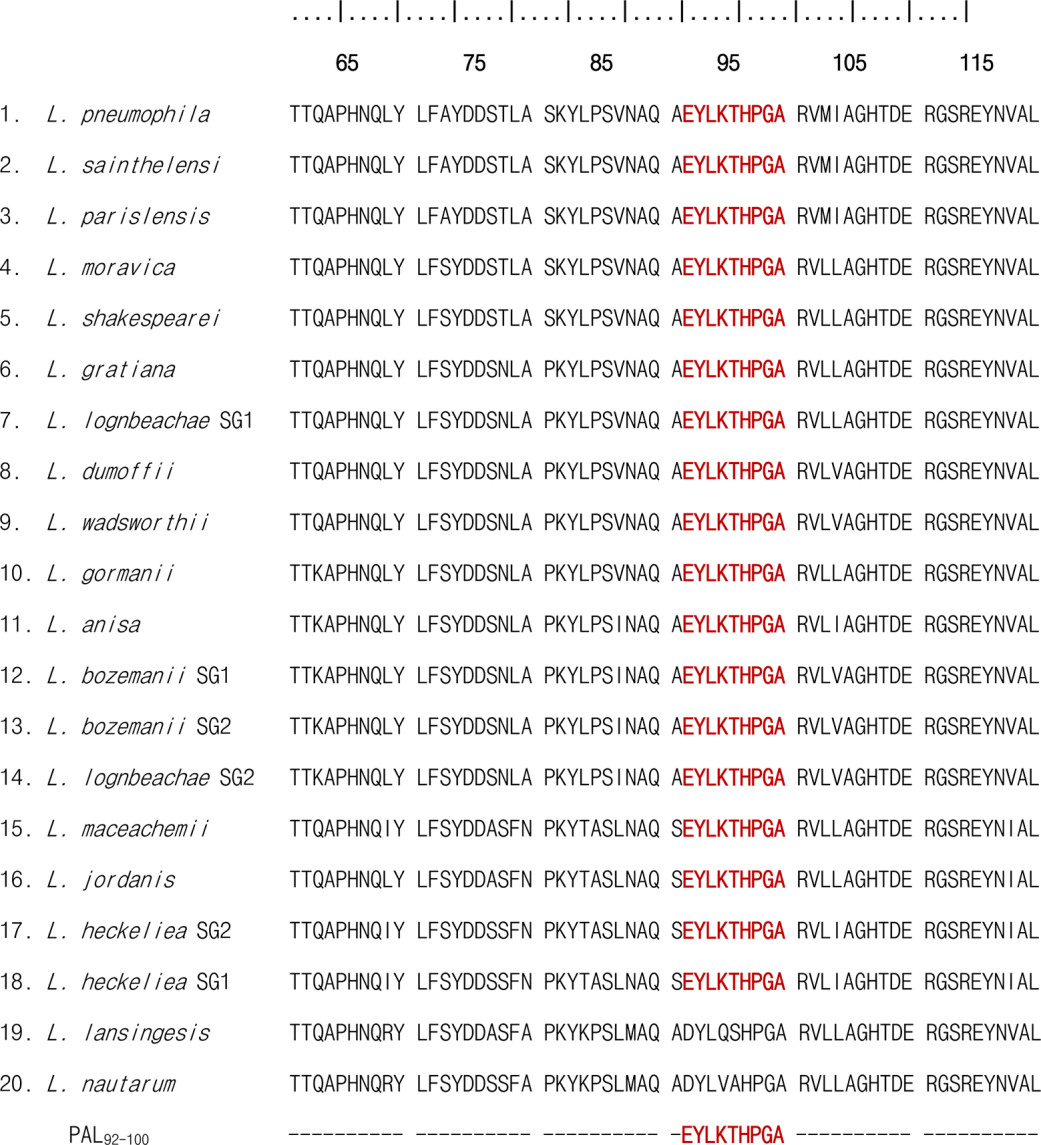
Multi-sequence alignment of the PAL sequence of *L. pneumophila* serogroup and 19 non-pneumophila *Legionella* species. Multi-sequence alignment of amino acid positions from 60 to 120 is shown. P8 (PAL_92-100_) peptide sequences are shared, as indicated by the red color.

To determine if the P8 (PAL_92-100_) peptide sequences of PAL proteins were similar among 20 *Legionella* species, we used the multiple alignment sequence program, CLUSTALW. As shown in **Figure 6**, the PAL_92-100_ peptide sequence was located in a conserved region in the PAL sequence of *L. pneumophila* and the genus *Legionella*, including *L. pneumophila* (ATCC 33152), *L. sainthelensi* (ATCC 33152), *L. parisiensis* (ATCC 35299), *L. moravica* (ATCC 43877), *L. shakespearei* (ATCC 49655), *L. gratiana* (ATCC 49413), *L. longbeachae* serogroup 1 (ATCC 33462), *L. dumoffii* (ATCC 33279), *L. wadsworthii* (ATCC 33877), *L. gormanii* (ATCC 33297), *L. anisa* (ATCC 35292), *L. bozemanii* serogroup 1 (ATCC 33217), *L. bozemanii* serogroup 2 (ATCC 35745), *L. longbeachae* serogroup 2 (ATCC 33484), *L. maceachemii* (ATCC 35300), *L. jordanis* (ATCC 33623), *L. heckeliea* serogroup 2 (ATCC 35999), *L. heckeliea* serogroup 1 (ATCC 35250), *L. lansingesis* (ATCC 49751), and *L. nautarum* (ATCC 49506). In this alignment, there was 100% homology in the PAL_92-100_ sequences between the *L. pneumophila* serogroup 1 and other *Legionella* species analyzed, with the exception of *L. lansingesis* and *L. nautarum*.

## Discussion

In this study, we determined that antigen-specific CD8^+^ T cells were mainly responsible for protection from *Legionella* infection in our PAL vaccine model. Our findings are somewhat consistent with those of previous published studies (9–11). In those studies, however, alveolar mononuclear cells (such as macrophages) were suggested to be effector cells against *L. pneumophila*. As we observed in this study, it is likely that cytokines (IFN-γ and TNF-α) released from PAL-specific CD8^+^ T cells may be also associated with resistance to *L. pneumophila* through activation of host’s mononuclear cells. Furthermore, our findings are fully compatible with those of a previous report indicating CD8^+^ T cells exert a major effector function in protection from infection with intracellular bacteria, such as *Rickettsia* and *Listeria monocytogenes* (29). In this process, CD8^+^ cytotoxic T cells kill infected cells by releasing granules (perforin and granzymes), as well as by granule-independent pathways.

In the present study, we also identified *Legionella* PAL-specific CD8^+^ T cell epitopes using three Class I MHC binding prediction programs, as well as IFN-γ and CTL assays. Out of the 13 predicted peptides, the P8 peptide, PAL_92-100_ (EYLKTHPGA) stimulated the greatest degree of IFN-γ production from the spleen cells of mice immunized with PAL DNA vaccines. Consistent with this finding, the PAL_92-100_ peptide stimulated PAL-specific CD8^+^ T cells as effector cells against target cells in an *in vitro* CTL assay. Moreover, the PAL_92-100_ peptide induced antigen-specific CTL activity in mice receiving co-immunization with a CpG-ODN adjuvant. Therefore, our findings support the notion that the PAL_92-100_ peptide is indeed an H-2K^d^-restricted CD8^+^ T cell epitope that can induce both INF-γ production from PAL-specific CD8^+^ T cells and CTL lytic activity *in vitro* and *in vivo*. Given peptide vaccines have been developed against various cancers and infectious diseases (30), we propose that Class I HLA epitopes of PAL proteins might be peptide vaccine candidates for protection from infection with *L. pneumophila* in humans.

We also demonstrated that the PAL_92-100_ peptide effectively induced protection against a lethal challenge with *L. pneumophila*. Mice immunized with the PAL_92-100_ peptide plus CpG-ODN had 100% survival after a lethal intravenous challenge with *L. pneumophila*, while all control animal groups died within several days after the challenge. Similarly, mice immunized with the PAL_92-100_ peptide plus CpG-ODN had 75–85.7% survival 14 days after a lethal intranasal challenge, as opposed to the control groups which had 0% survival after the challenge. The CpG-ODN adjuvant has been previously found to be an effective peptide vaccine adjuvant (24). The survival data were consistent with the bacterial burdens in the lungs of infected mice: bacterial counts were significantly lower in the lung tissues of the groups immunized with PAL_92-100_ peptide plus CpG-ODN than in the control groups (non-immunized mice and mice immunized with either the PAL_92-100_ peptide or CpG-ODN). Moreover, the IFN-γ and TNF-α concentrations produced by cultured splenocytes from cyclophosphamide-treated immunosuppressed mice were significantly greater in the groups immunized with PAL_92-100_ peptide plus CpG-ODN than in the control groups secreting basal concentrations of cytokines. Given the immunosuppressed mice were treated with 150 mg/kg cyclophosphamide, we first speculated that little, if any, protective immunity might be induced in these mice after immunization with the PAL_92-100_ peptide plus CpG-ODN. In the immunosuppressed animals, however, immunization with the PAL_92-100_ peptide not only increased survival rates after lethal infection with *L. pneumophila*, but also reduced bacterial burden in the lungs of infected mice. This result was consistent with lung pathology data indicating almost normal status after immunization with the PAL_92-100_ peptide plus CpG-ODN. Here it is highly likely that PAL_92-100_-specific CD8+ T cells are directly associated with protection from lung tissue damage resulting from *Legionella* infection. This is based upon the fact that PAL_92-100_-specific CD8+ T cells alone were inducible by this immunization scheme. However, this needs to be demonstrated by measuring the infiltration and functional status of CD8+ T cells in the lung tissues. These results suggest that the PAL_92-100_ epitope can induce a strong CTL response, thus leading to the eradication of intracellular *L. pneumophila* and normalization of lung tissues even in immunosuppressed animals. Our results underscore the possible utility of PAL vaccines for protection against *L. pneumophila* in elderly patients with weakened immunity. Taken together, our findings indicate it is highly likely that PAL_92-100_ epitopes induce antigen-specific CD8^+^ CTLs, thereby exerting protective activity against *L. pneumophila*. In addition, we found the PAL_92-100_ amino acid sequences of PAL proteins were highly conserved among serogroups of *L. pneumophila* and other *Legionella* species. In an international survey, *L. pneumophila* accounted for about 85 to 90% of cases of Legionnaires’ disease, but other *Legionella* species were also implicated in human infections (31). Therefore, it is plausible that the PAL_92-100_ peptide, as well as the native PAL protein, may be effective at inducing protective immunity against various *Legionella* species. On the other hand, we observed in our therapeutic study that the PAL_92-100_ peptide had no therapeutic activity against *L. pneumophila* (data not shown). This result might be ascribed to the short-term survival (*i.e*., 4 days) in the mice after bacterial challenge. Within this short interval, the PAL_92-100_ peptides were unlikely to stimulate antigen-specific CTL responses which were essential for anti-bacterial activity. Moreover, cyclophosphamide treatment required for this mortality study might have inhibited immune induction by PAL_92-100_ peptides. It is also possible that prompt administration of PAL_92-100_-specific CD8^+^ CTLs (generated *ex vivo*) to *Legionella*-infected animals may engender therapeutic activity against *L. pneumophila*. However, this theory needs to be tested. Taken together, this result suggests that the appropriate timing and magnitude of induction of antigen-specific CD8^+^ T cells may be a key factor in the development of protection against *L. pneumophila*.

In conclusion, to our knowledge, this is the first study to demonstrate that PAL_92-100_-specific CD8^+^ CTLs play an important role as effector cells in the host defense response against *L. pneumophila* in infected mice. Furthermore, *Legionella* PAL containing a well-conserved epitope might be useful as a vaccine against infection with various *Legionella* species.

## Data Availability Statement 

The original contributions presented in the study are included in the article/supplementary material; further inquiries can be directed to the corresponding author.

## Ethics Statement

The animal study was reviewed and approved by the Institutional Animal Care and Use Committee (IACUC) of the College of medicine, Korea University.

## Author Contributions

MJK developed the concept and the design of the study project, and directed the project. SJK carried out the experiments. J-IS encouraged SJK to undertake additional protection assays and supervised the findings of this work. SJK drafted the manuscript. All authors contributed to the article and approved the submitted version.

## Funding

This work was supported by a National Research Foundation of Korea (NRF) grant funded by the Korean government (Ministry of Science and ICT) (No. NRF-2017R1D1A1B03034363).

## Conflict of Interest

The authors declare that the research was conducted in the absence of any commercial or financial relationships that could be construed as a potential conflict of interest.
